# How Can We Study the Evolution of Animal Minds?

**DOI:** 10.3389/fpsyg.2016.00358

**Published:** 2016-03-15

**Authors:** Maxime Cauchoix, Alexis S. Chaine

**Affiliations:** ^1^Institute for Advanced Study in ToulouseToulouse, France; ^2^Station for Experimental Ecology in Moulis, CNRSMoulis, France

**Keywords:** cognitive ecology, natural selection, heredity, brood parasites, fitness cost, individual differences, path analysis

## Abstract

During the last 50 years, comparative cognition and neurosciences have improved our understanding of animal minds while evolutionary ecology has revealed how selection acts on traits through evolutionary time. We describe how cognition can be subject to natural selection like any other biological trait and how this evolutionary approach can be used to understand the evolution of animal cognition. We recount how *comparative* and *fitness methods* have been used to understand the evolution of cognition and outline how these approaches could extend our understanding of cognition. The fitness approach, in particular, offers unprecedented opportunities to study the evolutionary mechanisms responsible for variation in cognition within species and could allow us to investigate both proximate (i.e., neural and developmental) and ultimate (i.e., ecological and evolutionary) underpinnings of animal cognition together. We highlight recent studies that have successfully shown that cognitive traits can be under selection, in particular by linking individual variation in cognition to fitness. To bridge the gap between cognitive variation and fitness consequences and to better understand why and how selection can occur on cognition, we end this review by proposing a more integrative approach to study contemporary selection on cognitive traits combining socio-ecological data, minimally invasive neuroscience methods and measurement of ecologically relevant behaviors linked to fitness. Our overall goal in this review is to build a bridge between cognitive neuroscientists and evolutionary biologists, illustrate how their research could be complementary, and encourage evolutionary ecologists to include explicit attention to cognitive processes in their studies of behavior.

## Introduction

Niko Tinbergen (Tinbergen, 1963) proposed that biologists should try to understand animal behaviors in the light of two different and complimentary perspectives: the *proximate* and *ultimate* (see Laland et al., 2011; Bateson and Laland, 2013 for recent updates). While both approaches have been employed in the study of animal cognition, most studies have done so independently with little integration across fields. Indeed, even the language used in these two fields has diverged: “behavior” in cognitive sciences refers to the specific motor response to a cognitive test whereas “behavior” to evolutionary ecologists, the definition we follow in this review, refers to complex responses to social and ecological situations (e.g., mate choice, predator alarms, etc…) that often are an amalgamation of many different cognitive abilities. After some promising, integrative studies in the 1980s and 1990s (see Kamil, 1998 for a review), the last decades have seen the establishment of entirely independent lines of research with only a few notable exceptions. We now have a deeper understanding of how animal minds work, but we know very little about the evolution of or ecological pressures that shape cognition. Consequently, we know very little about what role cognition, a collection of highly plastic and flexible traits, plays in adaptation and biological evolution. We believe the time is ripe for evolutionary ecology studies to explicitly integrate cognition to generate a much stronger understanding of how the mind evolves.

**FIGURE 1:**
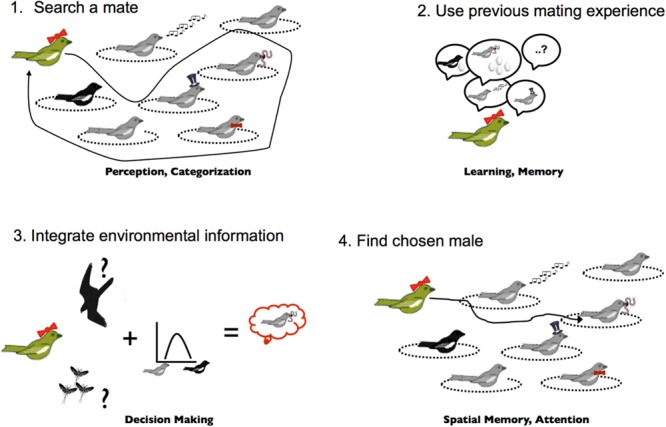
**Mate choice and cognitive capacities that could hypothetically play a role.** In bi-parental breeding songbirds, choosing an appropriate mate according to available male stock, previous breeding experience and actual environmental conditions is a behavior that will have drastic fitness consequences for any female and that is likely to rely on the interplay between various cognitive functions. Recognition of ornaments linked to different male qualities (e.g., good genes, parental care, nest defense, etc.) uses perception (visual and auditory) to detect male signals and categorization to group and identify male quality according to their ornaments (1). The use of previous breeding experience relies on past learning linking male ornaments and reproductive success from previous experiences (2). Mate choice itself, integrates all information available to the female including current ecology, mate options, and past experience supposedly through decision-making mechanisms (3). Finding the chosen mate, once the decision has been taken, probably relies on spatial memory to relocate the territory defended by the chosen male and endogenous attention to detect the chosen male from among the background of other males and environmental features (4).

Proximate studies focus on the mechanisms underlying given behaviors and the developmental biology of key structures. What stimuli trigger behaviors? How do neurons in the brain encode stimuli and transform them into behavior? What is the ontogeny of behavior? In other words, the proximate approach tries to understand how animal minds work. The current view for cognitive neuroscientists is that the animal mind emerges from brain activity as the neural machinery encodes, manipulates, stores and recalls information, which is together called ‘*cognition.*’ Cognition emerges when the brain transforms information into mental constructs or representations (Roitblat, 1982; Gallistel, 1990; Barsalou, 2014). For *cognitive scientists*, cognition is a synonym of ‘mind,’ which, operationally, is divided in various *cognitive functions.* Each function is implied in a specific step of information processing and thus plays a major role in ecologically relevant behaviors studied by behavioral ecologists (see also **Figure 1**). For readers not familiar with these functions, we define them here quickly highlighting the role they could play in the behavior of wild animals. *Perception* (i.e., vision, olfaction, audition, gustation, and somesthesia) contributes to the process by which mental representations are built from environmental stimulation. Most behaviors that are at least partially triggered by external stimuli rely on perception. Mate choice for instance necessitates first seeing or hearing a conspecific and recognizing it as a potential mate. The same thing is true for detecting prey or a predator. *Learning* is the ability to associate previously unrelated mental representations. Learning is ubiquitous in the behavior of wild animal. Even behaviors that have long been thought of as innate such as mate preference and imprinting seem to partially depend on learning (Verzijden et al., 2012). *Memory* is the ability to store mental representations either for a small amount of time (short term memory), a large amount of time (long term memory) or in relation to a particular on-going task (working memory). Short-term memory can be used to avoid already used food patches during foraging. Long-term memory plays a crucial role for animals that cache food in fall for winter use or for choosing a breeding habitat in migrating species according to experience in previous years. Working memory might be important during parental care for distributing food to all offspring despite disturbance. *Attention* is the mechanism allowing an individual to focus on only some mental representations among many. Attention can be exogenous, when a representation suddenly appears in the environment, for example during predator detection, or it can be endogenous when an animal is internally searching for one particular representation such as during foraging for specific prey. *Decision-making* is the process enabling an individual to compare mental representations and choose the most appropriate given the environmental context. Decision-making probably enters in the process of choosing a mate or a habitat among other things. Finally, *executive functions* (flexibility, categorization, problem solving etc…) enable an individual to perform operations and manipulations of mental representations. Flexibility, the ability to inhibit a previous association between representations to form a new one, appears crucial during dispersal to a new environment, choice of food in dynamic environments, and many social interactions. Categorization, the capability to group similar representations together, probably enables recognition of new kind of predators or preys. Problem solving, which is less well defined, is probably used by species living in urban environment or that use tools for foraging. Cognition is also sometimes divided according to the nature of the representation; one can for instance talk about spatial or social cognition. Homing or food caching relies on spatial cognition while discovery of new food patches or new foraging technics by observing conspecifics implies a certain level of social cognition.

The association between studies in psychology and neurosciences along with the advent of powerful new neuroimaging techniques [e.g., *In vivo* electrophysiology, Magnetic Resonance Imaging (MRI), Positron emission Tomography (PET), optogenetic etc.] has lead us to better understand how cognitive functions are linked to neural structures and neural activity in several animal species including humans. Despite this in depth understanding, much less progress has been made in understanding the evolutionary processes that have led to the patterns of cognition that we see (but see De Kort and Clayton, 2006).

Ultimate approaches focus on the evolutionary history of behaviors or traits and the selective pressures that favor the evolution of those traits. Those using this approach have focused on behaviors with only a few rare studies examining cognition *per se* (Bond and Kamil, 2002, 2006; Théry and Casas, 2002; Lyon, 2003). *Evolutionary biologists* and *behavioral ecologists* have been primarily interested in the ecology and evolution of behavior without examining the cognitive mechanisms underlying these behaviors. What ecological or social contexts are responsible for the evolution of a specific behavior? What role does evolutionary history (inheritance from a common ancestor) play in the evolution of that trait? What are the costs and benefits of behaviors and what do they imply for selection on the animal’s life history strategy? To answer these questions behavioral ecologists have adopted the Neo-Darwinian theoretical framework and developed tools and models to understand the extreme variability of behaviors within and among species. However, this approach focuses on the aggregate outcome of cognition and action (i.e., the behavior) and has usually considered the animal mind, or cognition, as a black box (Giraldeau, 2004). Indeed, much of behavioral or evolutionary ecology theory is based on strategic decision-making. While in some cases these strategic decisions reflect physiological trade-offs, many more cases reflect decisions made probably on the basis of processing external information gathered by an individual. Attention in such studies is placed on the quality of information and the outcome of a decision, but there is little understanding of how information is processed and how cognitive abilities enhance or constrain decisions based on the available information (but see Rowe, 1999, 2013; Stevens et al., 2005). For example, social behavior, individual recognition, mate choice, parental care, dispersal, foraging, and predator avoidance nearly always rely on gathering external information. How well an individual gathers that information, how well it remembers that information, and how it integrates different sources of information all depend on cognitive capacities – but these are rarely examined in studies of behavior. To illustrate this notion (**Figure 1**) we can imagine a female who must choose the best mate among males that each display a number of ornaments linked to various qualities (e.g., good genes, parental care, nest defense, etc…). A behavioral ecologist would examine the outcome of female choice behavior – compare males who were chosen relative to those who were not – to understand how male traits evolve. But female behavior is determined by a number of cognitive functions. How does a female integrate the information provided in each of the male’s sexual signals with information about the external ecological environment (e.g., are there many nest predators)? As the female comparison shops for the best male, how many of the males can she remember? If she chooses to return to the second male she saw, will she remember where he is and will she recognize him? This example illustrates just some of the cognitive processes related to one behavior that would have fundamental consequences for sexual selection theory. Many other behaviors and life history strategies will similarly depend on cognitive capacities and actual measurement of cognitive performances has the potential to fundamentally alter our views of behavior.

Our understanding of cognition and neurosciences could benefit from increased integration with evolution. The evolutionary history of species can help reveal how different cognitive functions or neural structures come about and can reveal constraints in the evolution of more complex cognition. Furthermore, understanding how selection in a natural setting acts on cognition and neural structures could reveal why specific abilities or mechanisms arise in some systems but not others. Understanding the evolutionary and ecological significance of cognition has been a major challenge in biology as highlighted in several recent books (Heyes and Huber, 2000; Dukas and Ratcliffe, 2009; Shettleworth, 2010) and review articles (Real, 1993; Kamil, 1998; Healy and Braithwaite, 2000; Dukas, 2004, 2008; Pravosudov and Smulders, 2010; Boogert et al., 2011; MacLean et al., 2012; Thornton et al., 2012, 2014; Pravosudov and Roth, 2013; Rowe and Healy, 2014; Croston et al., 2015; Morand-Ferron and Quinn, 2015) and has led to a new field of research called *cognitive ecology*. We argue that two factors will help significantly advance our understanding of animal cognition: (1) proximate and ultimate studies should develop lines of research that allow direct integration of the two fields and (2) that evolutionary studies begin to apply their research methods to cognition *per se* along with the behaviors that result from cognitive processes. In doing so, we will gain a better understanding of how cognitive systems evolve and how cognitive structures and function relate to the problems they evolved to solve.

Here, we first argue that brain and cognition are subject to natural selection just like any other biological trait. We then review past work testing popular hypotheses for cognitive evolution using comparative methods and highlight future directions to exploit using these methods. After this, we illustrate how measuring selection on cognition within a species provides a great opportunity to better understand the evolution of cognition. These two methods differ in the time scale of evolution that they measure (past vs. current) and each provides advantages and disadvantages. We then continue by presenting two lines of research as case studies—food hoarding and brood parasitism—that, in our view, have best integrated ecological challenges, natural behavior and underlying cognitive adaptation and which could serve as examples for future cognitive ecology research. We finish by arguing how more integrative studies can help us to understand how and why neurocognitive traits can be under selection in contemporary populations.

## Brain and Cognitive Functions are Subject to Natural Selection

To show that animal cognition evolves under direct natural selection requires that the three necessary conditions for selection and evolution that Darwin (1859, 1871) outlined apply to cognitive functions and brain (Dukas, 2004). Traits, or in this case cognitive functions, will evolve if (1) there is variability in cognition between individuals, (2) that this variability in cognitive performances is heritable, and (3) that this variation is related to variance in fitness (survival, reproductive success) under specific environmental conditions. Few studies have tackled these questions specifically, but evidence from the literature supports the notion that cognition *should* evolve under selection.

### Variation in Neurocognitive Ability

Inter-individual variability in animal cognition studies is rarely reported, yet without variation, cognition can not evolve. Studies in animal cognition generally focus on a small number of individuals because of the time involved in training and testing subjects and this small sample size precludes useful estimates of variation in cognitive performances. However, a recent meta-analysis of variation in individual performances at three common cognitive tasks for different species revealed very high inter-individual variability (Thornton and Lukas, 2012). Individual performances varied almost continuously from 25 to 100% success at a task in tests for species with the largest sample sizes. Some of this variation is influenced by age, sex, developmental conditions, or previous experience, so determining the extent of variation due to additive genes rather than plasticity will require large sample sizes at single cognitive tasks.

Despite little direct evidence, there are a number of indirect measures of cognitive variability that further support the notion that intraspecific variation in cognitive performances should be widespread. A growing number of recent studies focus on intraspecific variation in brain size including both within and among population variation (for a review see Gonda et al., 2013). This variation is also apparent in humans where inter-individual variation in brain structure and function has often been considered “noise” until recently (Kanai and Rees, 2011). Perhaps the best evidence of inter-individual cognitive variation comes from research on “general intelligence” in humans, which has been extensively documented through the use of intelligence or ‘IQ’ tests and shows high variation among individuals (Deary et al., 2010). Recent work has sought to tie variation between IQ in humans to its neural substrate (Deary et al., 2010; Penke et al., 2012).

### Heritability of Neurocognitive Abilities

Heritability of traits is difficult to measure since many non-genetic effects (common environment, parental care, maternal effects, etc…) contribute to resemblance between parents and offspring. For example, twin studies show that brain structure or function (e.g., face recognition) is heritable in humans (Peper et al., 2007; Wilmer et al., 2010; Zhu et al., 2010), yet non-genetic effects that occur in utero can not be excluded (but see Trzaskowski et al., 2013). One of the most powerful approaches to demonstrate that heritability of cognitive traits exists is through artificial selection experiments where species show phenotypic changes in response to researcher imposed selection criteria. Mery and Kawecki (2002, 2003, 2005) have shown that associative learning abilities for choice of oviposition substrate can be inherited in *Drosophila melanogaster* (see Kawecki, 2010 for a review;). Marked differences in learning and memory were shown between high learning and low learning selected *Drosophila* populations over 15 generations. Artificial selection of brain size in guppies (*Poecilia reticulata*) also suggests heritability of brain size (Kotrschal et al., 2013a) with a divergence in relative brain size of 9% between lines selected for large vs. small size over just two generations. Interestingly, large-brained females outperformed small-brained females in a numerical learning test, which also provides evidence for an association between increased brain size and higher cognition. These results should be treated cautiously since disentangling true heritability from plasticity would require more than two generations and a relaxation of selection to see if brain size differences persist (but see Healy and Rowe, 2013; Kotrschal et al., 2013b). Finally, the use of genome wide association has recently been used to demonstrate a genetic basis of human general intelligence and cognition. This approach has shown that a substantial proportion (between 40 and 66%) of individual differences in human general intelligence is linked to genetic variation (Davies et al., 2011; Benyamin et al., 2013; but see Chabris et al., 2012; Deary et al., 2012; Plomin et al., 2013).

However, estimating heritability of brain or cognitive traits in wild animal populations remains an exciting challenge (Croston et al., 2015).

### The Fitness Benefits of Cognition

Selection on cognitive abilities will occur if there are fitness benefits to particular cognitive phenotypes under a given set of environmental conditions. Addressing this question is challenging because it requires both an estimate of cognitive performance or brain structure/activity of a large number of individuals as well as fitness estimates, such as reproductive success or survival, for the same individuals. Most cognitive tests are run under laboratory conditions to control confounding effects on cognition and yet the best estimates of fitness benefits should be measured in the wild where the importance of a specific cognitive ability will also depend on the environmental context. Fitness measured in artificial selection experiments on cognition or brain size have reported costs and benefits of improved cognitive performances in insects (Dukas, 2008; Kawecki, 2010) or increased brain size in fishes (Kotrschal et al., 2013a), but the value of these traits in nature are unknown. In humans, general intelligence is correlated with school achievement, job performance, health, and survival (Deary et al., 2010), but not necessarily actual fitness (i.e., number of lifetime offspring that reproduce).

## Phylogenetic Comparative Studies of Brain and Cognition Evolution

Current tests of factors that influence the evolution of cognition have largely relied on comparative methods. The phylogenetic comparative approach (Felsenstein, 1985, 2008; Grafen, 1989; Harvey and Pagel, 1991; Ridley and Grafen, 1996; Felsenstein and Felenstein, 2004) allows us to ask questions about how the evolution of a trait occurs through comparison of extant species (although fossil evidence can be incorporated) while taking into account shared ancestry estimated from a phylogeny. We can then ask questions such as what factors (e.g., social or ecological) are associated with the evolution of a trait in the past (e.g., brain size), if that trait evolves directionally, how much common ancestry constrains evolution, and how the evolution of a trait influences speciation rates. Two major drawbacks to this approach are that causality cannot be determined given the correlative nature of the analyses and that past environments, and therefore past selection pressures, must be inferred from present conditions which may not always be a valid assumption.

The three major hypotheses of neurocognitive evolution that have been proposed focus on identifying primary factors that have driven differences in brain size and cognitive function across species. The first set of hypotheses suggest that cognition has evolved due to the value of *ecological intelligence*; the ability to find and extract food (Parker and Gibson, 1977; Byrne, 1997), manage high spatiotemporal variation in food resources (Sol et al., 2005), or manage and defend large territories (Clutton-Brock and Harvey, 1980). The second set of hypotheses propose that cognition has evolved primarily due to its value in *social intelligence*; the ability to negotiate and succeed through dominance in large groups (Whiten and Byrne, 1988; Dunbar, 1998) or alternatively the ability to manage positive relationships and social partnerships (Dunbar and Shultz, 2007, 2010; Emery et al., 2007). The third hypothesis, recently proposed to reconcile ecological and social drivers, suggests that cognition evolved to *buffer* individuals against environmental challenges by producing appropriate behavioral responses in new socio-ecological contexts (Allman and Hasenstaub, 1999; Deaner et al., 2003; Sol, 2009).

Each of the above hypotheses has been tested using comparative methods and each has found some support. For example, brain size depends on diet in mammals (Eisenberg and Wilson, 1978; Harvey et al., 1980; Gittleman, 1986; MacLean et al., 2014) suggesting a role for ecology. Likewise, brain size and neocortex size are related to social group size (Gittleman, 1986; Marino, 1996; Barton and Dunbar, 1997; Dunbar, 1998; Dunbar and Bever, 1998) and other metrics of social group structure in mammals (reviewed in Dunbar and Shultz, 2007) suggesting that social drivers are also important to the evolution of the brain and presumably cognition. Interestingly, comparison of ecological and social factors in ungulates, showed that relative brain size is influenced by both social and ecological factors while relative neocortex size is only influenced by sociality (Shultz and Dunbar, 2006). Finally, species with larger brains have been shown to survive better in novel environments (Sol et al., 2005, 2007, 2008) in support to the cognitive buffer hypothesis (Sol, 2009).

Comparative studies focused on brain size have also been largely criticized (Healy and Rowe, 2007; Roth et al., 2010a; Lihoreau et al., 2012). The high cognitive capacity of small-brained invertebrates, such as bees and ants, suggests that high cognitive capabilities do not require large overall brain size (Chittka and Niven, 2009). Initial research in comparative cognition used brain size as a coarse proxy for cognition, since morphological data for a broad variety of species is more readily available whereas finer scale measures of cognition or neurophysiology are not. However, measurements of brain size or brain structure volumes are too coarse grained given that current neuroscience methods enable us to study fine scale brain organization and function (Healy and Rowe, 2007; Roth et al., 2010a). For instance, cognitive neurosciences have revealed different brain networks and mechanism associated with different cognitive functions (Felleman and Van Essen, 1991; Grossberg, 2000; Fox et al., 2005; Meunier et al., 2009; Alivisatos et al., 2013; Smith et al., 2013; Vanduffel et al., 2014). Thus, instead of studying whole brain or neocortex size, comparative studies should focus on neural circuits and functioning that are known to be involved in the cognitive mechanism of interest when possible (Lihoreau et al., 2012).

Adaptive specialization of the hippocampus stands as one of the best examples illustrating how overall brain size *per se* could be a poor proxy for cognitive function. Storing and non-storing passerines do not differ in whole brain size or all spatial abilities but do differ in specific hippocampal structures and specific components of spatial memory (Krebs, 1990; Sherry et al., 1992; Clayton and Krebs, 1993, 1994a,b; Krebs et al., 1996; Shettleworth, 2003; Healy et al., 2009). Similarly homing pigeons have much larger hippocampus size compared to other wild and domestic pigeons (Rehkämper et al., 1988) as a result of artificial selection for spatial abilities (Rehkämper et al., 2008). As with storing passerines, only a specific part of the hippocampus seems to play a role in homing with distinctive roles of the left and right hippocampus linked to different components of homing behaviors in pigeons (Bingman et al., 2003, 2006; Kahn and Bingman, 2004; Bingman and Cheng, 2005; Gagliardo et al., 2005; Siegel et al., 2006). Unfortunately such detailed data on neural structures and the volume of specific brain regions is only available for a small number of species. Increasingly accessible neuro-cognitive imaging tools will soon allow phylogenetic comparative studies to focus on scales finer than brain size.

Efforts to address the problem that cognitive abilities cannot accurately be summarized solely by brain size have been made along two lines of comparative research: (i) spontaneous records of cognition-based behaviors (e.g., innovation) in the wild and (ii) comparative psychology experiments in the lab. The first line of research, also called ‘taxonomical counts of cognition in the wild’ (reviewed in Lefebvre, 2011), enables the study of large samples of “spontaneous” behavior occurring in the selective environment or at least a natural or semi-natural habitat. This approach has confirmed that relative brain size increases with increased tool use and frequency of innovation in birds (Lefebvre et al., 1997, 2004) and primates (Reader and Laland, 2002; Lefebvre et al., 2004), social learning in primates (Reader and Laland, 2002), or deception in primates (Byrne, 2004).

Taking the second approach, a few studies have begun comparing specific cognitive tasks among a small number of related species that differ in social or ecological conditions. One of the most advanced research programs of this kind, has been conducted on North American corvids (Balda et al., 1996; Kamil, 1998; Balda and Kamil, 2002) using a large number of cognitive tests run in the lab. Corvid species that rely heavily on food storing in the wild, such as Clark’s Nutcrakers (*Nucifraga columbiana*), typically outperform other corvids in tasks requiring spatial cognition (Olson et al., 1995); on the other hand, corvid species that are highly social, such as Pinyon Jays (*Gymnorhinus cyanocephalus*), are better in cognition tasks mimicking social challenges such as those designed to evaluate social learning, behavioral flexibility or transitive inference (Templeton et al., 1999; Bond et al., 2003, 2007, 2010). Studies in primates have similarly addressed how social structure is related to the evolution of cognitive performances. Comparing species that differ in their degree of sociality, Amici et al. (2008) have shown that species with fission-fusion social organization outperform species with very stable social groups in cognitive tasks requiring inhibitory control and/or flexibility. Very recently, one of the most accomplished studies merging phylogenetic and experimental cognition methods draws a slightly different picture (MacLean et al., 2014). MacLean and his 57 collaborators realized the feat of gathering cognitive performances of 36 animal species (from birds to rodents to apes) in two problem solving tasks measuring self-control. Their results suggest that the major proximate mechanism underlying the evolution of self-control is the absolute brain volume rather than residual brain volume corrected for body mass. They also report a significant relationship between cognitive performance and dietary breadth but not social organization in primates. Thus, this massive comparative cognition study challenges both the proxy of cognition (relative brain size) and the hypothesis (social brain hypothesis) tested in many brain size comparative studies and illustrates the danger of over interpreting comparative cognition studies. Continued efforts to link specific cognitive functions to their ecological and social settings present a promising avenue to understand the evolution of cognition while recognizing that different cognitive abilities may evolve under different environmental contexts. Doing comparative analyses on specific cognitive functions could become possible with the advent of increasingly automated test devices for measuring cognitive function to help build the sorts of across species datasets needed (Steurer et al., 2012; Morand-Ferron et al., 2015b).

A number of new directions using the comparative method have still not been sufficiently exploited. First and foremost, analyses should begin to compare specific regions of the brain, neural structure, or brain function rather than coarse measures of brain size. The increasing ease of using new technology (e.g., MRI, PET) to measure brain structures, connectivity, and function that are frequently measured in cognitive neurosciences could provide new insights on the link between the evolution of cognitive processes and ecological or social factors that influence cognition. Use of the comparative method on neural structure itself is generally lacking but could provide a very perspective on how the mind might evolve (Brenowitz and Zakon, 2015). Second, only a small range of questions using comparative methods have been addressed (see MacLean et al., 2012 for a review). For example, comparative methods can be used to examine the sequence of events in coevolution such that we could ask if the increase of a cognitive performance generally precedes or succeeds specific social or ecological changes. Likewise, we could examine the relative rates of evolution during the increase or decrease of a particular cognitive function. When we have measures of multiple cognitive structures or functions, we can also ask if phylogenetic patterns suggest an evolutionary trade-off between cognitive capacities, if they are independent, or if they evolve together. Indeed, mixing levels could allow us to ask if specific cognitive abilities usually coevolve with specific neural structures. Finally, we can ask how shifts in cognition are associated with the speciation process itself (Nicolakakis et al., 2003). Does the evolution of increased cognitive ability facilitate speciation? The biggest drawback to comparative methods, of course, is that it is correlative, thus preventing explicit tests of causation. Yet it provides an excellent tool to generate hypotheses that can then be tested in contemporary evolution studies. Comparative methods are also based on a number of key assumptions that may not always hold. We assume that the underlying phylogeny is true, that past environments and traits are similar to present ones, and that evolution is parsimonious which may not be valid for highly labile traits. Still, the comparative approach is unequaled in the breadth of taxonomic diversity and depth of evolutionary time.

## Intraspecific Selection on Neurocognitive Traits: The *Fitness* Approach

**FIGURE 2:**
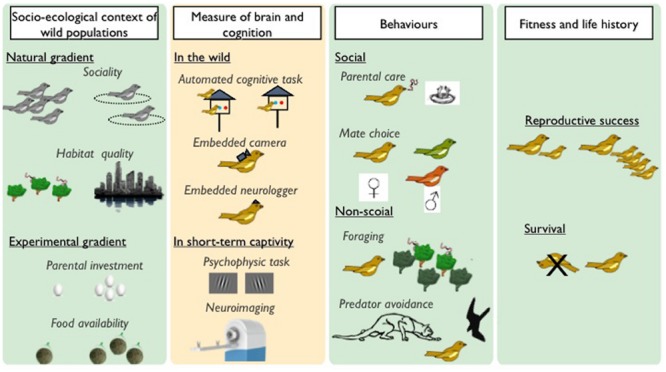
**How to study brain and cognition selection?** Ideal studies looking at contemporary selection on neurocognitive traits should integrate socio-ecological conditions **(left)**, neurocognitive traits **(middle left)**, ecologically relevant behaviors **(middle right)**, and fitness **(right)** variables. Such an approach seeks to truly merge behavioral and evolutionary (green background) and cognitive neuroscience (yellow background) methods. As examples: *Socio-ecological contexts of selection* could correspond to natural gradients in sociality (i.e., Population density, gregariousness), habitat quality (i.e., level of fragmentation, urbanization) and/or distribution of resources (i.e., harshness of the environment). Experimental manipulations of ecological factors, such as variation in food supplementation or reintroduction in a novel environment, are of particular interest to isolate ecological causes of selection. *Cognitive abilities* can be measured in the wild using automated foraging tasks. Such methods rely on individual identification usually mediated by passive integrated transponders (PIT) tags. However, some cognitive functions are difficult to measure in the wild and one may want to have a better control on motivational state and environmental parameters. Short-term periods of captivity seem appropriate in such a framework and potentially enable us to use current psychophysics protocols and equipment developed in comparative cognition labs. Development of embedded cameras or microphones has the potential to reveal spontaneous cognitive capabilities like tool use, social cognition or vocal communication. Likewise, neurologgers or transmitters enable us to measure *brain activity* (electroencephalogram, single unit activity) in free ranging wild animals. Spatial and whole *brain measurement* could also be assessed using MRI or PET devices in short term scanning protocol. *Ecologically relevant behaviors* linked to fitness such as parental care, mate choice, foraging, predator avoidance, should be measured to evaluate interactions between agents of selection and between cognitive abilities that are hypothesized to underlie these behaviors. The *fitness benefit* is traditionally assessed through evaluation of reproductive success (number of offspring who breed) or a measure of survival. Behavior associated with reproductive success (i.e., mating, number of offspring born, parental care) can also be used as proxies of fitness.

**FIGURE 3:**
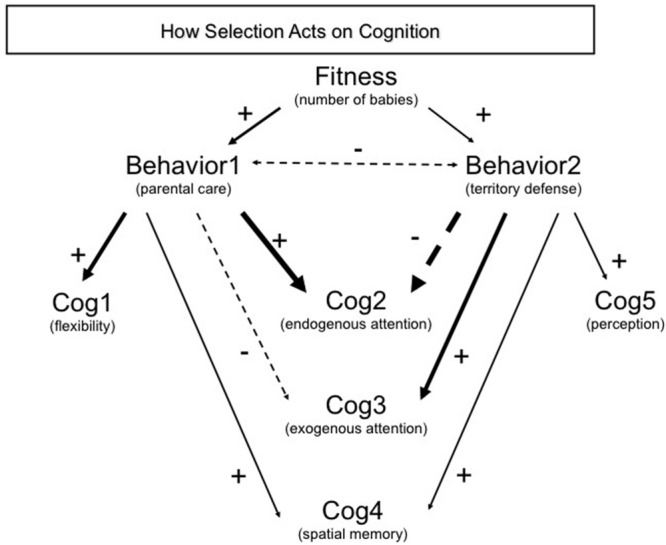
**Mapping the relationship between traits and how selection acts on cognition: a path approach.** Schematic example of a generic, hypothetical path analysis linking fitness (number of babies) that depends on two behaviors (parental care and territory defense), which in turn are dependent on a number of cognitive abilities. Arrows show the direction in which selection acts with solid arrows showing a positive relationship and dashed arrows a negative relationship and the thickness of arrows represents the strength of the relationship (partial correlation coefficient). Note that the direction opposite the arrows should indicate effects that underlie the above measure; for example, Cog1 plays an important role in the expression of Behav1. In this example, each behavior is linked to a number of cognitive abilities, but in different ways. Each behavior is linked to one cognitive ability that is only associated with that one behavior (Cog1 and Cog5), but also three other cognitive abilities that are also linked to both behaviors (Cog2–4). Cognitive traits that influence both behaviors show different patterns: attention (Cog 2, 3) shows opposite patterns between the two behaviors whereas memory (Cog4) has a positive relationship with both behaviors. The resulting selection and evolutionary dynamics will reflect these patterns: intense positive selection on memory, but more muted selection on attention. Path models can also estimate the relationship between traits such as the negative relationship we illustrate here as a double headed arrow (correlation with no causation implied) between parental care behavior and territory defense often thought to be antagonistic due to the effects of testosterone on each behavior.

Measuring contemporary selection has proved a powerful approach to understanding the evolution of traits and this method could be readily applied to the evolution of cognition. The basic premise of this ‘*fitness*’ approach follows Darwin’s (1859) theory of evolution which suggests that short term selection is the primary cause of evolutionary change and speciation. Therefore a careful examination of selection can help us understand how a trait evolves. An important caveat with this approach, though, is that we assume contemporary selection that we measure is representative of selection pressures that have led to current expression of cognition in a species. Selection can come from a number of origins which largely fall under *natural selection*, which includes the effects of abiotic influences and interspecific interactions on survival and reproduction (Darwin, 1859; and modern synthesis in Huxley, 1942), or *social selection* (West-Eberhard, 1983; Lyon and Montgomerie, 2012), which includes selection due to intraspecific social interactions including the effects of mating behavior (i.e., sexual selection, Darwin, 1871) and kin cooperation (i.e., kin selection, Hamilton, 1964) among other intraspecific interactions. Both of these forms of selection could act on cognitive functions and their underlying neural basis as described above (Boogert et al., 2011; Morand-Ferron and Quinn, 2015). There are two distinct advantages to the fitness method relative to the comparative method for studying neurocognitive evolution. The first advantage is that studies of contemporary selection measure fitness costs and benefits of specific traits which can provide a close match with measurements of cognitive performances and brain mechanisms currently studied in animal cognition and neurosciences (**Figure 2**). Thus the fitness approach provides opportunities to integrate our proximate understanding of cognition with new findings on the ultimate causes of cognitive evolution. The second advantage is that examination of selection ideally includes identification of the agent of selection or the specific social or ecological challenges that favor a specific trait. Adopting this approach helps us acknowledge that there may be multiple factors that select for a given cognitive ability and in some cases these factors might not act in concert (**Figure 3**). Likewise, it makes clear that each species will require only a subset of all cognitive skills given their environment. Of course the added depth of such studies come at a cost relative to comparative studies of cognitive evolution: time intensive *fitness* studies limit the range of species studied and thus provide a narrower, if deeper, view of evolution across species.

Studies specifically employing the *fitness* approach to understand the evolution of cognition are quite rare, but increasing interest is feeding the burgeoning field of *cognitive ecology*. As such, we are far from drawing general conclusions from such studies, but recent examples do illustrate the promise of this approach. Two recent studies have finally succeeded in measuring fitness consequences of problem-solving abilities in wild populations of great tits (*Parus major*; Cole et al., 2012; Cauchard et al., 2013). Cole et al. (2012) took birds into short term captivity to perform an innovation task to get food. Birds who solved the task had larger clutch sizes, but tended to desert their nest more often if disturbed (Cole et al., 2012). Cauchard et al. (2013) conducted cognitive tests in the wild, where birds had to remove an obstacle that blocked access to their nestbox. Those who could solve the puzzle had higher survival of offspring to fledging. Both studies found individual variation in cognitive performance of birds (solvers vs. non-solvers), so selection should act on problem solving abilities. Fitness costs of higher cognition (e.g., higher desertion rates; Cole et al., 2012), could produce a trade-off that helps maintain variation in cognitive abilities among individuals. Such a result is an important insight into the evolution of cognition: multiple selection pressures on the same cognitive trait would drastically change their evolutionary dynamics (e.g., speed of evolution) and trajectories (sequence of trait values on the path to an optimum; Lande and Arnold, 1983; Schluter, 1996; Sinervo and Svensson, 2002). These results are very promising, and should be diversified to a much broader range of cognitive functions (e.g., memory, learning, flexibility, etc…) and expanded to measures of brain structure or function (**Figure 2**). Furthermore, following pioneering research linking food hoarding behavior and spatial memory (see Pravosudov and Roth, 2013 for a review), understanding why cognition evolves will also require us to directly link cognitive performance (e.g., memory) to ecological challenges that the animals face in their natural environment (e.g., finding a food store). This last point is critical because if there are correlations among different cognitive abilities then measurement of selection (i.e., higher fitness) on one ability could be due to correlational selection on a different cognitive trait that is the actual target of selection (Lande and Arnold, 1983).

The above examples provide evidence for selection of cognitive abilities in wild animals, so the next big challenge for cognitive ecology is to identify which cognitive functions are critical for a species in their natural environment. While for most species we are still at the point of forming hypotheses on which cognitive abilities are critical (as we did for mate choice in **Figure 1**), there are a few studies that have moved well beyond this stage. Here we present two lines of research as examples of successfully linking natural behavior, cognitive function and ecological agents of selection. These studies contain elements of the fitness approach, but neither is complete: the first example lacks measures of fitness whereas the second has not studied cognitive abilities *per se*. Still, they illustrate how elements of the fitness approach can lead to new discoveries and insights into how cognition evolves.

## Case Studies

### The Evolutionary Ecology of Spatial Memory

Food hoarding animals rely on food caching and later retrieval of caches to survive winter and should have evolved excellent spatial memory abilities and associated neural structures (i.e., hippocampus). Based on this simple ecology-driven hypothesis, a flourishing literature on the cognitive ecology of food storing has emerged over the last 30 years. This work has successfully combined proximate and ultimate understandings of spatial cognition and serves as an example for future studies of the evolutionary ecology of cognition (see Brodin, 2010 for an historical review).

The first studies of the evolutionary and ecological significance of spatial memory employed the comparative framework, with the prediction that scatter food hoarding species should surpass non-hoarding species in spatial memory tasks and should have a relatively bigger hippocampus. However, results from these early studies were equivocal and difficult to interpret. The superiority of spatial capabilities in hoarding species was not always clear (reviewed in Healy et al., 2009). Furthermore, and more concerning, the comparative approach suffers from a number of confounding factors, such as morphological differences between species, that could never clearly be separated from performance in cognitive tasks [but see Kamil, 1998 for methods].

Problems with comparative analyses have been very elegantly solved by focusing on intra-specific variation in a number of landmark studies comparing populations exposed to different ecological contexts. In one of the earliest of such studies, Pravosudov and Clayton (2002) demonstrated that black-capped chickadees (*Poecile atricapilla*) living in harsh winter climates (i.e., Alaska) cache more food, have higher spatial memory capabilities, and have a larger hippocampus that contains more neurons than individuals of the same species in populations from milder climates (i.e., Colorado). While the appearance of adaptation is clear, such differences could reflect either local adaptation shaped by natural selection or result from plasticity in brain structure and behavior generated from the local environment. The persistence of among population differences in brain structure and caching behavior in common garden experiments, during which 10 days-old chicks from these different populations were hand-raised in identical environmental conditions, strongly argues for a role of natural selection in shaping local adaptation for spatial memory, neural density, and neurogenesis in the hippocampus (Roth et al., 2010b, 2012).

Recent analyses using this within species comparative approach in this and other species have further pushed our understanding of the links between cognition and evolutionary ecology and between proximate and ultimate understandings of cognitive evolution. Research in mountain chickadees (*P. gambeli*) along an altitudinal gradient has shown similar patterns of differentiation in food storage, spatial memory, and hippocampal characteristics as with contrasted populations in the black-capped chickadee (Freas et al., 2013). Other studies have extended this work on spatial memory differences across populations in caching behavior to differences between behavioral strategies within a population (LaDage et al., 2013). In side-blotched lizards (*Uta stansburiana*), males adopt one of three different mating strategies that rely to different degrees on spatial memory for territory defense and the distribution of available females across territories. Accordingly, characteristics of the dorsal cortex and hippocampus show differences among genetically determined alternative male strategies within a population (Ladage et al., 2009; LaDage et al., 2013). Work on hippocampal size contrasts among populations has recently been extended by fine scale studies of neural structure (Roth et al., 2010a, 2012) and differential gene expression (Pravosudov et al., 2013) within the hippocampus among contrasted populations of birds. The next step should be to measure the influence of spatial cognition and the underlying hippocampal structures or function on fitness in these contrasted environments.

### Cognitive Mechanisms of Host–Parasite Arm Races in Brood Parasites

Avian brood parasites lay their eggs in the nest of other individuals from the same or different species to avoids the costs of parental care but imposes a cost on the host (reviewed in Davies, 2011). These reciprocal selection pressures have often led to an arms race of detection and mimicry in egg appearance – a true cognitive battleground even if these studies do not measure specific cognitive functions. Studies of avian brood parasitism provide measures of selection on cognitive traits (recognition, rejection, deception), clear identification of the agent of selection, examination of how cognition influences the coevolutionary arms race, and neural traits associated with host–parasite life history.

Studies of avian brood parasitism have done an outstanding job of quantifying the fitness costs and benefits to each player of the host–parasite arms race—often linked to recognition of parasites (Lyon and Eadie, 2008; Davies, 2011). A parasite’s fitness is so intricately tied to acceptance by hosts that they must adapt to new host defenses either by identifying and changing to a new host or surpassing host defenses. Hosts, on the other hand, pay a cost of parasitism, but the evolution of new defenses (largely relying on cognitive functions such as perception and executive functions) must be balanced against the frequency of parasitism and the costs of producing better defenses (Rothstein, 1982; Davies and Brooke, 1988, 1989a,b; Lotem, 1993; Lotem et al., 1995). Costs of new defenses include developing the cognitive or morphological structures for new defenses as well as the added risk of expressing those defenses (e.g., rejecting own eggs), and these costs influence the evolution of recognition abilities. Plasticity in host recognition reveals the importance that making an incorrect choice can have for the evolution of egg rejection. For example, some common cuckoo hosts avoid rejecting their own eggs (recognition error) when parasites are not present by only increasing rejection rates when adult cuckoos are seen in the vicinity of the nest (Davies and Brooke, 1988). In South American coots, intraspecific parasitism leads to egg rejection, but an interspecific parasite, the blackheaded duck, that imposes no parental care costs is only rejected when ecological conditions render incubation more costly (Lyon and Eadie, 2004). Globally, studies of avian brood parasites have provided an excellent understanding of the selective environment generated by host–parasite interactions that influences the evolution of recognition and rejection of eggs.

Mimicry-recognition-rejection arms races reveal the link between cognitive abilities and the evolutionary dynamics of host–parasite systems. Arms races in avian brood parasites related to egg mimicry push host recognition systems to identify parasites while avoiding recognition errors (Rothstein, 1982; Davies and Brooke, 1988; Lotem, 1993; Lotem et al., 1995; Rodríguez-Gironés and Lotem, 1999). The accuracy of identifying a mimetic egg depends on visual discrimination abilities and recent studies have begun to specifically integrate this process using ‘visual modeling’–information on cone sensitivity and objective measures of egg color patterns–to understand rejection behavior, or the lack thereof, in some species (Cassey et al., 2008; Spottiswoode and Stevens, 2010). Recent findings show that visual detection of parasites can improve by integrating multiple sources of information (Spottiswoode and Stevens, 2010) a hallmark of complex decision-making. Egg cues (Langmore et al., 2009; Spottiswoode and Stevens, 2010; Svennungsen and Holen, 2010; de la Colina et al., 2012), external cues of parasite presence (Davies and Brooke, 1988), or counting the number of eggs laid (Lyon, 2003) have all been shown as means to improve the decision to reject parasite eggs. Use of multiple and disparate cues to improve the accuracy of rejection behavior would require executive functions to weigh these different criteria in a rejection decision and future research could examine this cognitive ability. Not all host species reject eggs or chicks, which implies that physiological or cognitive limitations may also influence the detection of a parasitic egg (Rothstein, 1982; Davies and Brooke, 1988; Lotem, 1993; Lotem et al., 1995; Rodríguez-Gironés and Lotem, 1999), but studies on the cognitive aspects of this pattern are currently lacking.

An understanding of the cognitive mechanisms underlying rejection have also played an important role in understanding why despite close visual mimicry in eggs, nestlings are rarely mimetic. One hypothesis is that unlike egg recognition where comparisons between multiple host eggs and a single parasitic egg makes discrimination possible, having only a single parasite chick in the nest (e.g., common cuckoos) could have severe long term fitness costs if birds *learn* the appearance of their chicks (Lotem, 1993). Indeed, learning does seem to play a role in identification and discrimination of eggs (Rothstein, 1974, 1978; Strausberger and Rothstein, 2009) and possibly chicks (Shizuka and Lyon, 2010; Colombelli-Négrel et al., 2012). A possible solution in some species, such as the North American coot, might be to use extra cues such as hatch order and soft rejection (e.g., lower feeding) to help identify parasitic chicks while reducing the risk of mis-imprinting (Shizuka and Lyon, 2010, 2011). In essence, behavior may mitigate the fitness effects of constraint on cognitive functions such as recognition accuracy. These models and empirical results show that the cognitive mechanisms underlying how a species is able to recognize its eggs and chicks plays an important role in the evolution of the host-parasite arms race. In turn, quantifying the fitness effects of recognition and decision-making under a complex ecological context shows what selective pressures shape cognitive abilities and the impact of cognitive constraints.

Finally, a few studies have also begun to investigate the link between neurophysiology and the ecology of brood parasites. Initial studies focused primarily on whole brain size or hippocampus size in brood parasites and their non-parasitic relatives since each species should face different ecological imperatives. Generally, whole brain size tends to be smaller in brood-parasites than their closest relatives (Iwaniuk, 2004; Overington, 2011; Corfield et al., 2013), which could be linked to less complex cognitive function needed in the absence of parental care in brood parasites (Boerner and Krüger, 2008). Hippocampus size, however, varies predictably with the need for excellent spatial memory in brood parasites. Brood parasites have an enlarged hippocampus in the breeding season (Clayton et al., 1997), the sex that searches for nests tends to have a larger hippocampus than the other sex (Sherry et al., 1993; Reboreda et al., 1996), and brood parasites have a relatively larger hippocampus than closely related non-parasites (Reboreda et al., 1996; Corfield et al., 2013). Furthermore, recent analysis has uncovered a specific region of the hippocampus that is enlarged in parasitic species relative to others (Nair-Roberts et al., 2006), suggesting brain regions may have evolved to manage the specific needs of brood parasites relative to other spatial memory. These studies provide a rare example of direct linkage between ecology and neurophysiology on a well understood fitness landscape. An exciting next step in such systems could be to examine variation in neural structure with variation in the ability of different hosts – either across or within a species – to reject parasitic eggs or chicks.

The above studies provide some of the best examples of how discrimination ability links with cognitive decision making under natural ecological conditions. While many of these host-parasite studies have not specifically been framed in terms of cognitive ecology, the focus on discrimination, recognition, learning, and decision making are all clearly linked to cognition and could further link to both specific cognitive abilities studied in other organisms and to neurophysiological studies. Together with the strong understanding of the fitness costs and benefits of host-parasite coevolution, these systems provide an excellent opportunity to link cognition, neurophysiology, and evolutionary biology.

The above examples illustrate the power of the fitness approach in linking ecological context to the evolution of cognitive abilities and neural structure. The focus on the fitness costs and benefits of specific abilities could provide added insight into the evolutionary dynamics of cognitive traits, why certain cognitive traits do not evolve, how variation in selection pressures contribute to the evolution of cognition (**Figure 3**), and under what conditions specific cognitive abilities should be favored. Of course the fitness approach does have some drawbacks that will undoubtedly limit its reach in the short term. Fitness studies are very time intensive and resource hungry, yet usually provide information on only one species in one context. Gathering sufficient information on multiple environments and across species will take time. Furthermore, fitness studies focus on contemporary selection and must assume that current conditions are representative of conditions during the evolution of the focal traits from simpler versions or from different versions in other species. Still, the ability to disentangle effects that contribute to the evolution of cognition (**Figure 3**) and experimentally test hypotheses to determine causative effects make the fitness approach an under-utilized method that would nicely complement comparative studies. Below, we outline some of the major areas where we think this fitness approach can provide the most interesting contributions to our understanding of cognitive evolution.

## Toward Integrative Studies of Natural Selection on Neurocognitive Traits

First steps have been taken to develop a new line of research in the evolution of cognition by looking at contemporary selection in wild animal populations (Keagy et al., 2011; Cole et al., 2012; Cauchard et al., 2013; Isden et al., 2013; Thornton et al., 2014; Morand-Ferron and Quinn, 2015; Morand-Ferron et al., 2015a). However, as with every nascent field, many issues have yet to be addressed. Some of the issues raised so far are that we still need to develop psychometric methods and new technologies to measure cognition in the wild (Thornton et al., 2014; Morand-Ferron et al., 2015b; Shaw et al., 2015), we need to deal with confounding variables such as motivation or personality (Rowe and Healy, 2014; Morand-Ferron et al., 2015a), and we need to show consistent individual differences in cognition (Griffin et al., 2015; Morand-Ferron et al., 2015a). Furthermore, variation in cognition may not necessarily have fitness consequences in some species or some populations, so we need sufficient sample sizes to determine when cognitive variation is important to fitness and therefore evolving (Rowe and Healy, 2014).

We believe that it is now time to increase integrative studies of contemporary selection of cognition. In addition to measuring specific cognitive functions and fitness, a number of related lines of inquiry have already been highlighted by others. To give just one example, quantitative genetics and measures of heritability will be critical to understanding how cognition will evolve, the degree to which genes and the environment affect cognition, and how genes related to different cognitive capacities interact and coevolve (Croston et al., 2015; Morand-Ferron et al., 2015a). Recent advances in gene expression (Young et al., 1997; Young and Wang, 2004) and gene modification (Kelly and Goodson, 2014; Dance, 2015) also provide new tools to understanding the genomic basis of cognition and how selection acts at this level. Many other related lines of inquiry will need to be developed, and below we highlight a few that we feel are most urgent.

Here we highlight three challenging topics in fitness studies of cognition that we believe will provide important insight to cognitive neurosciences (**Figure 2**). First, development of minimally invasive psychophysics and neurosciences methods could help integrate neurosciences with the fitness approach to draw parallels and contrasts between traditional lab-based cognition studies and cognition in the wild. Second, we need a better understanding of the ecological and social agents of selection that acts on cognition. In other words, what ecological problems have specific cognitive traits evolved to solve? Finally, integration of ecologically relevant behaviors that influence fitness and life history in wild populations with cognitive functions that underlie these behaviors could prove critical in linking how selection acts on variation in cognition.

### Integration of Cognitive Neurosciences Methods

Adaptive specialization in brain structure and cognition has been successfully investigated at the population level by sacrificing birds and using common garden experiments (Pravosudov and Roth, 2013). However, a totally different approach has to be taken for one interested in measuring selection on cognition and the brain. Studying the fitness consequences of individual differences in neurocognitive traits implies interfering as little as possible with wild animals and thus, requires the development of new methods and approaches (**Figure 2**).

The lack of exchange between cognitive scientists and behavioral ecologists has left a big gap between methods used to study contemporary selection on cognition in the wild (Keagy et al., 2009; Cole et al., 2012; Cauchard et al., 2013; Isden et al., 2013) and current methods used in psychophysics or comparative cognition (Fagot and Bonté, 2010; Steurer et al., 2012). The first studies to bridge this gap have mainly been carried out using problem-solving tasks, for which underlying cognitive and neural mechanisms remain vague (Healy, 2012; Rowe and Healy, 2014; Thornton et al., 2014) or using custom made mechanical tasks which require a lot of manipulation by experimenters and therefore only allows tests on a limited number of individuals (Isden et al., 2013). Use of psychometrics methods has been proposed, but not yet implemented in studies in the wild (Thornton et al., 2014). Such an approach would move the field toward more standardized measures of cognitive functions. However, this approach also clearly implies the use of new hardware to collect cognitive performances in wild animals (Morand-Ferron et al., 2015a). The first step in that direction has been taken by Morand-Ferron et al. (2015b) who managed to run a fully automated associative learning task on 80 free ranging tits equipped with passive integrated transponders (PIT tags) using portable operant boxes. The next step to reach current standards of cognitive testing in comparative cognition (Fagot and Bonté, 2010; Steurer et al., 2012) would be to develop systems equipped with touch screen displays and therefore the possibility to run virtually any of the classical psychophysics or cognitive task developed by psychologists. Likewise, development of embedded cameras or microphones could reveal spontaneous cognitive capabilities like tool use, social cognition, or vocal communication (Rutz et al., 2007; Krause et al., 2013; Anisimov et al., 2014).

To our knowledge, no study has yet examined contemporary selection directly on brain structure or function of wild animals. And yet, new recording methods to collect neural activities in wild animals open many opportunities to launch a research line neurocognitive selection. Mini embedded EcoG (electrocorticogram) recorders, called neuro-loggers, have been developed to track neural activities of free ranging wild animals. This method has been successfully adapted to different species: pigeons (Vyssotski et al., 2006, 2009), ostrich (Lesku et al., 2011), sloths (Rattenborg et al., 2008), sandpipers (Lesku et al., 2012), or owls (Scriba et al., 2014). The study on sandpipers illustrates that such tool can be used to link brain activity recorded in wild animals, for instance using individual brain rhythm characteristics, to fitness (Lesku et al., 2012). Linking such measurements with cognitive tests and/or measures of behavior and fitness would provide unique insight into selection on brain function in natural environments.

Measurements of cognitive performance or neural function in the wild can be challenging, but an initial approach conducting such measures on wild animals during short term captivity might represent a start in the right direction. Captivity allows for control of motivation through food deprivation and offers standardized environmental and social conditions to each individual. Such an approach has been used to link cognition in captivity with fitness in the wild (Cole et al., 2012) and to characterize differences in cognition between populations in common garden experiments (Pravosudov and Roth, 2013). Likewise, non-invasive MRI or PET methods also appear as very promising tool to study selection on neural structure and function (Van der Linden et al., 2009; Cross et al., 2012, 2013; Marzluff et al., 2012; De Groof et al., 2013). Recent work in birds has generated MRI brain atlases for a number of species (Poirier et al., 2008; De Groof et al., 2013, 2015; Güntürkün et al., 2013), and could be used to more easily examine variation among individuals in neural structure and function in wild birds during short term captivity. However, for linking individual differences to fitness, these approaches in captivity are not ideal since stress induced by captivity is likely to affect fitness, but also cognitive performance (Pravosudov, 2003). Despite this drawback, these methods do provide a solid first step in the right direction of studying selection on cognition in the wild.

### Integrating Ecological and Socio-Ecological Contexts of Selections

To better understand when and why cognition is under selection, it is critical to identify the *agents of selection*, by measuring possible social and ecological contexts of selection along with fitness (**Figure 2**). In other words, identifying the agents of selection on a cognitive ability allows us to understand under what ecological or social conditions you would expect the evolution of specific capacities. In turn, this helps us better understand cognitive processes through the problems they are meant to solve. Indeed, it is quite possible that what we measure as a specific cognitive ability in many different systems (e.g., memory), might actually serve different functions in each system and therefore we might anticipate differences in the details of how such memory works. For example, memory in food hoarding birds might operate differently than spatial memory in brood parasites since the accuracy and number of items to recall may be very different. Likewise, selection can vary across time (Chaine and Lyon, 2008; Siepielski et al., 2009), and focus on specific agents of selection on cognition can help us understand the causes of temporal fluctuations in selection and their evolutionary consequences (Bro-Jørgensen, 2010; Kingsolver et al., 2012). By identifying the agents of selection, we gain insight into why differences might exist in cognitive performances between species, between populations of a single species, and between individuals within a population (e.g., between genders, between individuals playing different strategies (Reboreda et al., 1996; LaDage et al., 2013). Furthermore, it allows us to understand the ecological context of cognition, why particular cognitive traits evolve, and may help us identify nuances within a specific cognitive ability that evolved in two different contexts.

Equally important to identifying agents of selection is the ability to exclude potential agents of selection that might act on a cognitive trait. Some have argued that cognitive variation does not necessarily have fitness consequences (Rowe and Healy, 2014), and supporting this notion would also require showing that selective agents logically linked to that cognitive ability does not act. By identifying which contexts do or do not cause selection on a cognitive ability, we can understand when and where specific traits will evolve (**Figure 2**). Socio-ecological contexts of selection could correspond to natural gradients in sociality (e.g., population density, gregariousness, social strategy distribution), habitat quality (e.g., level of fragmentation, urbanization) and/or distribution of resources (e.g., food or nest site availability, harshness of the environment). Experimental manipulations of ecological factors, such as variation in food supplementation or reintroduction in a novel environment are of particular interest to isolate ecological causes of selection and an important tool in excluding certain hypothesized agents of selection.

Most cognitive abilities intervene in a broad array of contexts and behaviors, so understanding selection on and the evolutionary dynamics of cognitive abilities requires identifying and understanding all, or at least many, of these contexts. For example, spatial memory will play a role in recalling the placement of food, predators, potential mates, territory boundaries, and migration routes among other things, yet the importance of accuracy, benefits of forgetting, and fitness gained from each is likely different. Consequently, overall selection will reflect the importance of each task relative to fitness. The identification of a number of different agents of selection on a single cognitive trait, allows us to understand the complementary or conflicting effects that the environment can have on the evolution of cognitive abilities which often function in many different contexts (see next section; **Figure 3**).

### Integration of Behaviors and Cognition under Selection

The links between cognitive abilities, behaviors in the wild, and fitness can be intricate and therefore will lead to complex patterns of selection (**Figure 3**). Behaviors are usually what is exposed to selection from the ecological and social environment, yet the relationship between cognition and complex behaviors is not always straight forward making it difficult to directly link fitness and cognitive performances. The link between cognition and behavior matters because a given cognitive trait could contribute to a number of behaviors and if selection acts differently on each behavior, you will get very different selection dynamics on the underlying cognitive trait (Lande and Arnold, 1983; Schluter, 1996; Sinervo and Svensson, 2002). Likewise, if two cognitive traits act in concert to produce a specific behavior, you can get correlated evolution and even genetic coupling of those cognitive traits (i.e., linkage disequilibrium between genes coding different cognitive functions; Sinervo and Svensson, 2002). This problem is exacerbated for neural structures since a given neural structure likely underlies a number of cognitive abilities and each cognitive ability relies on a number of brain regions. Given that multiple cognitive abilities likely interact with multiple behaviors (**Figure 3**), we expect strong correlational selection effects to act on the evolution of cognition. Without clear mapping of different agents of selection on multiple behaviors as well as mapping the links between multiple behaviors and multiple cognitive abilities, we will have only a poor understanding of the evolutionary dynamics underlying cognition. Not doing so can lead to erroneous conclusions about whether selection is acting on a cognitive trait, how intense selection is on a trait, and how cognition evolves over longer time scales.

A useful approach to disentangle the multi-faceted interactions between behaviors, cognitive traits, and fitness would be to employ path analysis or structural equation modeling (Wright, 1934; Li, 1975; Thomas et al., 2007; Frank, 2013; Westland, 2015). Researchers define one or more hierarchical path models (e.g., **Figure 3**) and use their data to estimate the links between traits and their relationship with fitness (e.g., Sinervo and DeNardo, 1996; Svensson et al., 2001; Sih et al., 2002; Thomas et al., 2007; Frank, 2013). This provides a nice match to the problem at hand since in essence, mapping selection to cognition is more evocative of a network than a simple flow diagram making a multi-variate approach to link ecology to behavior to cognition is essential. Variation in space or time can also be examined by estimating alternative models with datasets that correspond to different scenarios (e.g., Sih et al., 2002), thereby specifically examining how selection might fluctuate or differ between populations. Finally, researchers can test alternative path models to better understand the relationships between cognitive traits, behaviors, and fitness using both observational data and experiments (Wootton, 1994; Sinervo and DeNardo, 1996). Indeed, manipulations (e.g., behavior, hormones, or gene products/mRNA that influence the cognitive trait) are a very important tool in disentangling the links between traits and such results can be directly used in a path framework. Overall, structural equation modeling provides an important tool to understand the complex relationships we expect between cognition, behavior, and selection and how these relationships will influence the evolution of cognition.

## Conclusion

We have highlighted two ways to investigate the evolution of cognitive processes in animals: the comparative approach focuses on evolutionary history while the fitness approach examines contemporary selection. Much of our knowledge on the evolution of cognition comes from the comparative approach and the full application of recently developed phylogenetic tools should allow for interesting new results in this line of research. However, since cognition presents all the characteristics of traits under selection (variation, heritability, and fitness benefits), we believe that taking the fitness approach to cognitive function will allow us to better explore the evolutionary mechanisms that shape animal minds. Furthermore, the fitness approach more easily allows us to integrate proximate and ultimate factors underlying animal cognition in a single study, as suggested 50 years ago by Tinbergen (1963) and to identify agent of selection. In both cases, understanding the natural context under which cognitive functions evolve, provides an interesting and different perspective to complement studies of cognition in model species held in captivity.

## Author Contributions

All authors listed, have made substantial, direct and intellectual contribution to the work, and approved it for publication.

## Conflict of Interest Statement

The authors declare that the research was conducted in the absence of any commercial or financial relationships that could be construed as a potential conflict of interest.
